# Human genetic factors associated with susceptibility to SARS-CoV-2 infection and COVID-19 disease severity

**DOI:** 10.1186/s40246-020-00290-4

**Published:** 2020-10-22

**Authors:** Cleo Anastassopoulou, Zoi Gkizarioti, George P. Patrinos, Athanasios Tsakris

**Affiliations:** 1 Laboratory of Microbiology, Medical School, National and Kapodistrian University of Athens, 75 Mikras Asias Street, 11527 Athens, Greece; 2 Laboratory of Pharmacogenomics and Individualized Therapy, Department of Pharmacy, School of Health Sciences, University of Patras, Patras, Greece; 3 Zayed Center of Health Sciences, United Arab Emirates University, Al-Ain, United Arab Emirates; 4 College of Medicine and Health Sciences, Department of Pathology, United Arab Emirates University, Al-Ain, United Arab Emirates

**Keywords:** Polymorphisms, Allelic variation, Genetic predisposition, Genotype, Clinical outcome, SARS-CoV-2, COVID-19, Coronavirus, X chromosome, TLR7

## Abstract

**Abstract:**

**Background:**

The emergence of the novel coronavirus in Wuhan, Hubei Province, China, in December 2019 marked the synchronization of the world to a peculiar clock that is counting infected cases and deaths instead of hours and minutes. The pandemic, highly transmissible severe acute respiratory syndrome coronavirus 2 (SARS-CoV-2) has indeed caused considerable morbidity and mortality and drastically changed our everyday lives. As we continue to become acquainted with the seventh coronavirus known to infect our species, a number of its characteristics keep surprising us. Among those is the wide spectrum of clinical manifestations of the resulting coronavirus disease 2019 (COVID-19), which ranges from asymptomatic or mildly symptomatic infections to severe pneumonia, respiratory failure, and death.

**Main body:**

Data, now from patient populations, are beginning to accumulate on human genetic factors that may contribute to the observed diversified disease severity. Therefore, we deemed it prudent to review the associations between specific human genetic variants and clinical disease severity or susceptibility to infection that have been reported in the literature to date (at the time of writing this article in early August 2020 with updates in mid-September). With this work, we hope (i) to assist the fast-paced biomedical research efforts to combat the virus by critically summarizing current knowledge on the potential role of host genetics, and (ii) to help guide current genetics and genomics research towards candidate gene variants that warrant further investigation in larger studies. We found that determinants of differing severity of COVID-19 predominantly include components of the immune response to the virus, while determinants of differing susceptibility to SARS-CoV-2 mostly entail genes related to the initial stages of infection (i.e., binding of the cell surface receptor and entry).

**Conclusion:**

Elucidating the genetic determinants of COVID-19 severity and susceptibility to SARS-CoV-2 infection would allow for the stratification of individuals according to risk so that those at high risk would be prioritized for immunization, for example, if or when safe and effective vaccines are developed. Our enhanced understanding of the underlying biological mechanisms could also guide personalized therapeutics. Such knowledge is already beginning to provide clues that help explain, at least in part, current epidemiologic observations regarding the typically more severe or benign disease course in older males and children, respectively.

## Background

Ever since the emergence of the novel coronavirus in Wuhan, Hubei Province, China, in December 2019, the world has been synchronized to a peculiar clock that is not counting hours and minutes, but infected cases and deaths. The pandemic for the past 4 months severe acute respiratory syndrome coronavirus 2 (SARS-CoV-2) has indeed caused considerable morbidity, mortality, and economic damage and drastically changed our everyday lives. As we continue to learn more about the seventh coronavirus known to infect our species, a number of baffling factors about it keep surprising us. Among those is the wide spectrum of clinical manifestations that characterizes the resulting coronavirus disease 2019 (COVID-19), which ranges from lack of symptoms (as perceived by individuals because measurable signs of infection such as viral RNA may be detectable from various anatomic sites, including the naso- or oro-pharynx and the gastrointestinal tract, even of asymptomatic persons), or mild symptoms of the upper respiratory tract to severe pneumonia with acute respiratory distress syndrome (ARDS) and death [1, 2]. Advanced age, male gender, and comorbidities, such as cardiovascular disease or hypertension, and also diabetes and obesity have been identified as risk factors for more severe COVID-19 [3, 4]. Still, which and to what extent specific genetic factors may account for the predisposition of individuals to develop severe disease or to contract the infection remains unclear.

Herein, we summarize and comment on the associations between specific human genetic variants and clinical disease severity or susceptibility to infection that have been reported in the literature to date (at the time of writing this article in early August 2020 with updates as of mid-September 2020). Our main objectives were twofold: (i) to assist current biomedical research efforts to combat the virus by presenting in a comprehensive manner what is known on the subject, and (ii) to help guide current genetics and genomics research towards candidate gene variants that warrant further investigation in larger studies. In turn, this knowledge could help stratify individuals according to risk, thus allowing for the prioritization of those at greater risk for protection, for instance, if or when safe and effective prophylactic vaccines or therapeutics are developed. Furthermore, we aspire that this accumulated knowledge could ultimately help open new avenues, ideally for innovative personalized treatments. Preliminary information of possible genetic determinants of COVID-19 was highlighted in a mini-review by Godri Pollitt et al. that was published in May [5]. To the best of our knowledge, this is the first comprehensive review on associations between specific genetic loci or genes, which may well differ based on their geographical distribution, and COVID-19. While revising this article, an exhaustive review of the literature was published by LoPresti et al. on host genetic factors related to coronaviruses that affect different species, including humans [6].

## Main text

### Literature search

We searched PubMed/MEDLINE for all English-language original articles or reviews reporting on potential associations between human genetic factors and susceptibility to SARS-CoV-2 infection or COVID-19 severity, up to August 12, 2020 (with updating as of September 11, 2020, during the revision of the manuscript). Articles on preprint servers (i.e., BioRxiv and MedRxiv) were included in our search. The search was performed using all combinations of terms related to the novel coronavirus and the disease (e.g., “SARS-CoV-2,” “2019-nCoV,” and “COVID-19”) on the one hand, and terms concerning susceptibility to infection or disease severity (e.g., “polymorphisms,” “allelic variation,” “genetic predisposition,” “genotype,” “clinical outcome”) as well as the names of individual genes in which relevant polymorphisms were found (e.g., “TLR7,” “ACE2”), on the other. All study types and countries of origin, irrespectively of the size of patients’ cohorts or whether positive or negative results were reported, were included in our analysis. Reference was not made to social and economic inequalities that tend to disproportionately affect populations of specific ancestries or ethnic backgrounds and increase risk mainly due to limited access to health care.

## Results

A summary of reported associations between human genes and differing severity of COVID-19 or differing susceptibility to SARS-CoV-2 that are discussed in detail below is presented in Table 1.

**Table 1:** Summary of reported associations between human genes and COVID-19

Gene(s)	Polymorphism(s)	Chromosome location	Reported COVID-19 associations	Reference(s)
*ABO*	rs657152	9q34.2	Higher risk of infection for blood group A vs. non-A (OR 1.45, 95% CI 1.20–1.75, *P* = 1.48 × 10^−4^) and lower risk of infection for blood group O vs. non-O (OR 0.65, 95% CI 0.53–0.79, *P* = 1.06 × 10^−5^)	[7, 38, 39]
*ACE2*	p.Arg514-Gly	Xp22.2	Cardiovascular and pulmonary conditions in the African/African-American population by altering AGT-ACE2 pathway	[49]
*ApoE*	rs429358-C-C (e4e4)	19q13.32	Severe disease independently of pre-existing dementia, cardiovascular disease, and type 2 diabetes	[32]
*HLA*	B*46:01 and B*15:03	6p21.33	Vulnerable to disease for HLA-B*46:01 and cross-protective T cell-based immunity for HLA-B*15:03	[15]
*IFITM3*	rs12252-C/C	11p15.5	Mild-to-moderate disease requiring hospitalization	[35]
*SLC6A20*, *LZTFL1*, *CCR9*, *FYCO1*, *CXCR6, XCR1*	rs11385942-GA	3p21.31	Severe disease (respiratory failure) (OR 1.77, 95% CI 1.48–2.11, *P* = 1.15 × 10^−10^)	[7]
*TLR7*	g.12905756_12905759del and g.12906010G>T	Xp22.2	Severe disease	[19]
*TMEM189-UBE2V1*	rs6020298-A	20q13.13	Severe disease	[17]
*TMPRSS2*	p.Val160Met (rs12329760)	21q22.3	Increased susceptibility to disease and for risk factors, e.g., cancer	[49]

### Determinants of differing severity of COVID-19

#### Locus 3p21.31 spanning genes SLC6A20, LZTFL1, CCR9, FYCO1, CXCR6, and XCR1

A genome-wide association study (GWAS) analyzed 8,582,968 single nucleotide polymorphisms (SNPs) from 1980 patients severely afflicted by COVID-19 from the Italian and Spanish epicenters of the pandemic in Europe and conducted a meta-analysis of the two case-control panels that included 835 patients and 1255 control participants from Italy and 775 patients and 950 control participants from Spain, respectively [7]. The study did not detect significant associations of severe disease defined as respiratory failure with a single causative gene, but rather with a multi-gene cluster on chromosome 3. Among those were the *LZTFL1* that is strongly expressed in human lung cells and the sodium–imino acid (proline) transporter 1 (SIT1)-encoding *SLC6A20* that functionally interacts with the cell-surface receptor of the novel coronavirus, angiotensin-converting enzyme 2 (ACE2) [8, 9]. The remaining genes of the locus encode chemokine receptors of the C-C and CXC families which are defined based on the position of the two conserved cysteine residues in the N-termini of these members of the superfamily of G protein-coupled receptors (GPCRs); chemokines control cell migration associated with immune surveillance by trafficking effector cells to sites of infection and inflammation [10]. *CXCR6*, for instance, regulates the specific location of lung-resident memory CD8^+^ T cells throughout the sustained immune response to airway pathogens, including influenza viruses, while flanking genes (e.g., *CCR1* and *CCR2*) also have relevant functions [11, 12]. The risk allele GA of the lead SNP rs11385942 was associated with reduced expression of *CXCR6* and increased expression of *SLC6A20* [7].

Associations within the same locus of ~ 50 kb at chromosome 3p21.31 that may have been inherited from Neandertals [13] were also reported by preliminary results from the COVID-19 Host Genetics Consortium that included both mildly and severely affected patients [14]. Ideally, the comparison of such a GWAS analysis that highlights associations rather than demonstrating causality for a specific genetic variant would be between hospitalized patients with severe disease and infected mildly symptomatic or asymptomatic individuals. Instead, population-based controls were included of people from the same geographic region who were not hospitalized, and, importantly, whose exposure to the virus is unknown. However, given the unprecedented conditions of the pandemic under which this research was conducted—and in such a short period of time—any potential limitations in design cannot diminish its significance.

Thus, the ~ 1.5 times significantly higher frequency of the risk allele among hospitalized patients who received mechanical ventilation than among those who received supplemental oxygen only, as well as the younger age of patients who were homozygous for the risk allele than patients who were heterozygous or homozygous for the non-risk allele, support the involvement of the chromosome 3p21.31 locus in COVID-19 severity and suggest that some of the protective benefits of younger age are trumped by a double dose [7]. Further studies are needed to explore the underlying biomechanistic details of these associations.

#### HLA-B*46:01 and HLA-B*15:03

Potential associations between the genetic variability in major histocompatibility complex (MHC) class I genes (human leukocyte antigen [HLA] A, B, and C) and the susceptibility to SARS-CoV-2 and severity of COVID-19 were investigated by Nguyen et al. who performed a comprehensive in silico analysis of viral peptide-MHC class I binding affinity across 145 HLA-A, HLA-B, and HLA-C genotypes for all SARS-CoV-2 peptides [15]. The fewest predicted binding peptides for SARS-CoV-2 were found for HLA-B*46:01, suggesting that individuals harboring this allele may be particularly vulnerable to COVID-19, as previously shown for SARS-CoV-1 [16]. Conversely, the greatest capacity to present highly conserved SARS-CoV-2 peptides that are shared among common human coronaviruses were detected for HLA-B*15:03, suggesting that this allelic variant could enable cross-protective T cell-based immunity [15]. Meanwhile, preliminary results from a study from China indicated that the HLA-A*11:01, -B*51:01, and -C*14:02 alleles significantly predispose patients for the worst clinical outcome [17]. Another recently published report from Italy added three more *HLA* alleles, HLA-DRB1*15:01, -DQB1*06:02, and -B*27:07, to the list that may predispose for a less favorable outcome by analyzing a group of 99 patients affected by a severe or extremely severe form of COVID-19 compared to a reference group of 1017 individuals [18]. Further studies are needed to clarify the role of single *HLA* alleles in COVID-19 severity.

### The X-chromosomal *TLR7*

Rare, putative loss-of-function variants of the X-chromosomal *Toll-Like Receptor 7* (*TLR7*) gene that were associated with impaired type I and II IFN responses were identified by rapid clinical whole-exome sequencing of the patients and segregation in available family members [19]. The study involved a case series of four young males (two pairs of brothers younger than 35 years from two unrelated families) with severe COVID-19 from the Netherlands [19]. A maternally inherited 4-nucleotide deletion (c.2129_2132del; p.[Gln710Argfs*18]) was identified in members of the first family that included a brother who died of the infection; the affected members of the second family carried a missense variant (c.2383G>T; p.[Val795Phe]) in *TLR7*. Downstream type I interferon (IFN) signaling was transcriptionally downregulated in primary peripheral blood mononuclear cells (PBMCs) from the patients compared with family members and controls, as measured by the significantly decreased mRNA expression of *IRF7*, *IFNB1*, and *ISG15* on stimulation with the TLR7 agonist imiquimod. The production of the type II IFN-γ was also decreased in patients in response to stimulation with imiquimod. Thus, *TLR7*, which is subject to selective evolutionary constraints against predicted loss of function [20], seems to be an essential component of the innate immunity against coronaviruses, including SARS-CoV-2 [21–23]. Nevertheless, SARS-CoV-2 presumably induces a lower antiviral transcriptional response, marked by low type I IFN levels and elevated chemokine expression, compared to other respiratory viruses [24].

Apart from discovering a new genetic link that could open a novel avenue for the exploration of potential treatments, this study possibly also provides an explanation for the observed trend of higher fatalities from COVID-19 in men than in women. Several immune-related genes and regulatory elements, such as non-coding micro RNA (miRNA), that are extensively involved in both the innate and adaptive immune responses are found in the X chromosome, the complexity of statistical analysis of which poses an obstacle to its inclusion in genome-wide and candidate association studies of infectious diseases [25]. However, apart from the influence of sex hormones and socioeconomic and behavioral factors, the X chromosome, X-linked genes, and X chromosome inactivation mechanisms contribute to sexual dimorphism. In other words, males are haploid for the X chromosome that they inherit from their mothers and, therefore, any abnormality in genes on the X chromosome is more likely to be expressed phenotypically and have more pronounced immunological consequences. In contrast, the fact that females carry both a maternal and a paternal X chromosome, and are thus functional mosaics for X-linked genes mainly due to X chromosome inactivation, could contribute to an immunological advantage for females in many infections, possibly including COVID-19. Females tend to have higher antibody responses, but also more adverse reactions in response to a number of vaccines and they are more prone to developing autoimmunity, including systemic lupus erythematosus [26–28]. Testosterone acts as an immune suppressor through the upregulation of anti-inflammatory cytokines (interleukin-10, IL-10), while estrogen enhances the immune system by upregulating pro-inflammatory cytokines (tumor necrosis factor alpha (TNFα)) [27, 29].

#### ApoE

Pre-existing dementia was also identified as a major risk factor (odds ratio [OR] = 3.07, 95% CI 1.71 to 5.50) for COVID-19 severity in older adults in the UK Biobank [30]. The *Apolipoprotein E* (*APOE*) gene has three major isoforms, APOE2, APOE3, and APOE4, that are encoded by e2, e3, and e4 alleles, respectively, which in turn are haplotypes of the SNPs rs429358 and rs7412 on chromosome 19 (T-T, C-T, and C-C, correspondingly) [31]. The *ApoE* e4e4 homozygous genotype was found to increase the risk of severe COVID-19, independently of pre-existing dementia, cardiovascular disease, and type 2 diabetes [32]. In addition to affecting lipo-protein function and subsequent cardio-metabolic diseases, the *ApoE* e4 allele moderates macrophage pro-/anti-inflammatory phenotypes [33]. *ApoE* is one of the highly co-expressed genes in type II alveolar cells in the lungs, where the ACE2 receptor that SARS-CoV-2 uses for cell entry is highly expressed [32]. Further studies could help unravel the biological mechanisms linking *ApoE* genotypes to COVID-19 severity.

#### IFITM3

The SNP rs12252-C/C in the interferon-induced transmembrane protein 3-encoding gene *IFITM3* that is linked to severe influenza [34] was detected in a patient from Wuhan, China, with mild-to-moderate COVID-19 that required hospitalization but recovered [35]. Τhis was a finding from a single case report; however, as the prevalence of this SNP is 26.5% in the Chinese population (the 1000 Genomes Project) [36], further investigation of the *IFITM3*-rs12252-C/C allele in larger cohorts of people with COVID-19 may be worth pursuing.

#### TMEM189-UBE2V1

A preprint of the first host genetic study in China analyzed a total of 22.2 million genetic variants by deeply sequencing 332 COVID-19 patients from the Shenzhen Third People’s Hospital who had been categorized by varying levels of severity to asymptomatic, mild, moderate, severe, or critical ill, after the correction of potential confounding factors. The most significant gene loci associated with disease severity were located in *TMEM189-UBE2V1* that is involved in the IL-1 signaling pathway [17].

### Determinants of differing susceptibility to SARS-CoV-2

#### ABO blood groups

As with SARS-CoV-1 [37], ABO blood groups have been implicated in susceptibility to SARS-CoV-2 infection as well. In particular, blood groups A and O have been associated with minimally increased and decreased risk of acquiring COVID-19 than non-A and non-O groups, respectively [7, 38, 39]. The biologic mechanisms underlying the potentially differing susceptibility to infection by ABO blood group may stem directly from the ABO blood group and the development of neutralizing antibodies against protein-linked N-glycans, for example, [40], or, indirectly, from other mediated effects that could include the stabilization of von Willebrand factor [41–45]. Zietz and Tatonetti [46] also found evidence for association between ABO and Rh blood groups, with depletion of O and enrichment of B blood groups among SARS-CoV-2-positive patients; moreover, Rh(D)-positive blood types were found to be associated with SARS-CoV-2 infection and death following infection, without confounding due to demographics or other known risk factors [46]. Nonetheless, the observed associations with blood types were not corroborated by the COVID-19 Host Genetics Consortium [14], suggesting they may be circumstantial, stemming from factors unrelated to COVID-19.

#### ACE2 and TMPRSS2

SARS-CoV-2 infection of susceptible cells is dependent on at least two host cell factors: the receptor ACE2 for cell entry, as already mentioned, and the transmembrane serine protease (TMPRSS2) for priming of the spike (S) protein of the virus [47]. Single-cell RNA sequencing has been used recently to investigate the distribution of *ACE2* expression, which together with *TMPRSS2* are thought to dictate viral tissue tropism [48]. A recently published comparative genetic analysis of approximately 81,000 human genomes across different populations suggested possible associations of coding region variants of *ACE2* and *TMPRSS2* with COVID-19 susceptibility, severity, and clinical outcomes [49].

*ACE2* polymorphisms were more likely to be associated with cardiovascular and pulmonary conditions by altering the angiotensinogen (AGT)-ACE2 interactions, such as p.Arg514-Gly in the African/African-American population [49]. Apart from the potential effects of differential polymorphisms on susceptibility and outcome in different ethnic populations, the localization of *ACE2* to the X chromosome may help explain the increased risk for males compared to females. As for the X-chromosomal *TLR7* gene, the mono-allelic versus the bi-allelic presence of this gene too may have an adverse impact on the natural history and prognosis of COVID-19 in males.

*TMPRSS2* is a key gene in prostate cancer, which, as an associated translocation, drives ETS-family oncogene expression in a large proportion of tumors [50]. The differential genetic susceptibility to COVID-19 as well as for risk factors, including those with cancer, may be explained at least in part by unique but prevalent polymorphisms, including p.Val160Met (rs12329760), an expression quantitative trait locus (eQTL) in *TMPRSS2*. The likely adverse effect of the rs12329760 polymorphism in the coding region of the *TMPRSS2* gene was corroborated by a recent study that used data of the 1000 Genome Project and web-based tools [51]. This study further identified 17 polymorphisms in the non-coding regions of *TMPRSS2* as well as an additional 31 such polymorphisms with possible functional consequences by providing binding sites for transcription factors and miRNAs, in the non-coding sequences of the following genes: three in *ACE2*, ten in *TMPRSS11A*, twelve in *neutrophil elastase* (*ELANE)*, and six in *cathepsin L (CTSL)* [51]. The oncogenic roles of *TMPRSS2* may be linked to poor outcomes with COVID-19 as well [52], while the localization of the gene on 21q22.3 could place individuals with Down syndrome at high risk for COVID-19 infection [49]. Interestingly, the developmental regulation of *TMPRSS2*, as suggested by the finding, by Schuler et al. [53], of the highest expression in ciliated cells and type I alveolar epithelial cells (AT1) that increased with aging in humans and mice, may provide the link of the relative protection of children from severe COVID-19. Hence, it may be worth pursuing further studies of age-related polymorphisms for *TMPRSS2*, using such cohorts as the Genetic Epidemiology Research on Adult Health and Aging (GERA) [54].

A study from Italy, the second epicenter of the pandemic after Wuhan, that examined exome and SNP-array data from a large Italian cohort in comparison to other Europeans and East Asians, found no evidence that *ACE2* is associated with disease severity or sex bias; on the other hand, *TMPRSS2* levels and genetic variants proved to be possible candidate disease modulators in that study, prompting for rapid experimental validations on large patient cohorts [55]. Another recently published study from Italy that mined whole-exome sequencing data of 6930 individuals from five different centers participating in the Network of Italian Genomes (NIG) identified a number of *ACE2* variants with a potential impact on protein stability [56]. Among those were three common missense changes predicted to interfere with protein structure and stabilization, p.(Asn720Asp), p.(Lys26Arg), and p.(Gly211Arg), as well as the rare variants p.(Leu351Val) and p.(Pro389His), which possibly interfere with the binding of the spike of SARS-CoV-2 or with the internalization process [57]. Exome sequencing and analysis of a smaller cohort of 131 COVID-19 patients from Italy for genetic variants of *TMPRSS2*, *PCSK3*, *DPP4*, and *BSG* genes identified 17 variants [58]. The statistically more frequent alleles found in patients compared to the EUR GnomAD reference population included a missense variant (c.893G>A) in *PCSK3* and c.331G>A, c.23G>T, and c.589G>A variant alleles in *TMPRSS2* [58]. Genetic variants of the *ACE2* gene from the same cohort of 131 Italian patients were also examined in comparison to 1000 individuals (500 males and 500 females) [57]. One intronic c.439+4G>A and two missense c.1888G>C p.(Asp630His) and c.2158A>G p.(Asn720Asp) with a similar frequency in male and female were found. However, only the c.1888G>C p.(Asp630His) variant, which was nonetheless detected in just one heterozygous COVID-19 patient, showed a statistically different frequency compared to the ethnically matched population [57]. The initial study of Chinese patients that is still in preprint, showed a decreasing allele frequency of the p.Val197Met missense variant which affects the stability of the TMPRSS2 protein, among the severely infected compared to the mildly infected patients and the general population [17].

Finally, a recent survey of the critical loci for host-viral interaction and entry into host cells (*ACE2* and *TMPRSS2*) or viral genomic RNA sensing (i.e., *TLR3/7/8*) showed that they are highly conserved in all populations, with very few non-synonymous variants [59]. The authors conclude that non-genetic factors, such as age, underlying medical conditions, and environmental risk factors like air pollution, are likely to contribute to differing susceptibility to SARS-CoV-2 infection [59]. Further studies are needed to confirm this conclusion or to offer support to detected potential associations with host genetics.

## Conclusions

The increasing availability of data from COVID-19 patient populations is allowing for potential associations to be made between specific gene loci and disease severity or susceptibility to infection. Components of the immune response to the virus seem to be principally related to the observed inter-individual variation in disease severity, while genes related to the binding of the ACE2 cell surface receptor and entry at the initial stages of infection, largely determine the differing susceptibility to SARS-CoV-2. Such evidence-based risk assessment could lead to personalized preventive measures and therapeutic options. Perhaps the time has come for precision medicine strategies that could help us survive, if not win, this tug-of-war with the novel coronavirus.

## Data Availability

Not applicable.

## Abbreviations

ACE2: Angiotensin-converting enzyme 2
AGT: Angiotensinogen
APOE: Apolipoprotein E
ARDS: Acute respiratory distress syndrome
AT1: Type I alveolar epithelial cells
CI: Confidence interval
CTSL: Cathepsin L
COVID-19: Coronavirus disease 2019
ELANE: Neutrophil elastase
eQTL: Expression quantitative trait locus
GERA: Genetic Epidemiology Research on Adult Health and Aging
GPCRs: G protein-coupled receptors
GWAS: Genome-Wide Association Study
HLA: Human leukocyte antigen
IFITM3: Interferon-induced transmembrane protein 3
IFN: Interferon
IL-10: Interleukin-10
MHC: Major histocompatibility complex
miRNA: Micro RNA
OR: Odds ratio
PBMCs: Peripheral blood mononuclear cells
SARS-CoV-2: Severe acute respiratory syndrome coronavirus 2
SNP: Single nucleotide polymorphism
SIT1: Sodium–imino acid (proline) transporter 1
S: Spike
TLR7: Toll-like receptor 7
TMPRSS2: Transmembrane serine protease
TNFα: Tumor necrosis factor alpha
